# Atorvastatin Treatment for Atrial Fibrillation Reduces Serum High-Sensitivity C-Reactive Protein Levels

**DOI:** 10.1155/2015/402481

**Published:** 2015-07-01

**Authors:** Fang-Cheng Su, Xi-Dong Li, Shao-Xia Sun, Ming-Yu Shi, Feng-Hua Xue, Shi-Chao Teng, Li Jiang, Jing Zhu, Feng Yin, Hong-Yue Gu

**Affiliations:** ^1^Department of Emergency, Weifang People's Hospital, Weifang 261041, China; ^2^Department of Cardiology, Linyi People's Hospital, Linyi 276003, China; ^3^Department of Cardiology, The First Affiliated Hospital of Harbin Medical University, Harbin 150001, China

## Abstract

We investigated whether serum hs-CRP levels predict the efficacy of atrial fibrillation (AF) treated with atorvastatin. Bibliographic databases were exhaustively searched for studies relevant to the research topic. Newcastle-Ottawa Scale (NOS) criteria, combined with the Quality Assessment of Diagnostic Accuracy Studies (QUADAS), were applied for study quality assessment. Our meta-analysis identified seven cohort studies (2006~2013), providing information on the change in serum hs-CRP levels in AF patients receiving atorvastatin therapy. After atorvastatin treatment, hs-CRP level in AF patients decreased significantly (SMD = 1.02, 95% CI: 0.58–1.47, *P* < 0.001). Subgroup analysis by country and hs-CRP detection methods suggested a negative relationship between atorvastatin treatment and hs-CRP levels among Chinese AF patients (SMD = 1.34, 95% CI: 1.00–1.69, *P* < 0.001) and by using ELISA method (SMD = 1.11, 95% CI: 0.51–1.71, *P* < 0.001), but not among Turkish population and using INA method (all *P* > 0.05). Egger's test showed no publication bias (*P* = 0.450). hs-CRP was clearly lowered in AF patients treated with atorvastatin, which may be helpful in the choice of statin agents for AF treatment. However, longer follow-ups are necessary to assess the clinical value of lowering hs-CRP in the clinical setting of AF treatment outcomes.

## 1. Introduction

Atrial fibrillation (AF) refers to extremely rapid and disorganized cardiac rhythm which may result in elevated afterload, increased filling pressures, and left atrial enlargement [1, 2]. The clinical manifestation of AF is a rapid heart rate usually associated with palpitations, exercise intolerance, anginal chest pain, and congestive heart failure [3]. The annual prevalence of AF per 1000 person-years is 1.9 in females and 3.1 in males under the age of 65. AF influences 5% of population over 65 years and 7.1% of octogenarians [2, 4]. The incidence of AF in the US is projected to reach 5.6 to 12 million in 2050. AF confers 1.5–2.0-fold greater relative risk of mortality [5, 6]. Clinically, electrophysiological abnormalities, surgical interventions, increase in atrial pressure, pharmacological drugs, inflammation or infiltrative atrial disease, cardiac atrium ischemia, and endocrine diseases may cause AF [7]. AF is a major public health problem and impairs patients' quality of life, and various antiarrhythmic drugs have been utilized in the clinical management of AF patients [8, 9]. In this context, AF patients treated with atorvastatin showed decreased levels of high-sensitivity C-reactive protein (hs-CRP), a protein produced by the liver during infection, tissue injury, and chronic inflammation, indicating that atorvastatin may have significant clinical benefits in AF treatment and in prevention of AF recurrence [9, 10].

Atorvastatin belongs to a class of drugs known as the statins, routinely prescribed to reduce blood cholesterol and to prevent adverse events related to cardiovascular diseases [11]. Statins inhibit the expression of tissue factors and cell adhesion proteins, prevent monocyte adhesion with the vascular endothelium and subsequently the subendothelial space, inhibit the release of inflammatory cytokines and the formation of foam cells, and decrease the levels of C-reactive protein (CRP) [7]. Atorvastatin, similar to the other statins, has been shown to reduce hs-CRP levels [12]. CRP is an acute-phase plasma protein that binds to complement proteins commonly assembled on apoptotic cells, on the surfaces of pathogens, and is implicated in the systemic response to inflammation [13]. CRP synthesis is rapidly elevated within hours after infection or tissue injury, indicating that it may be conducive to supporting host defense and participates in innate immune response [14]. CRP and SP pathways converge due to the fact that inflammation, endothelial/endocardial dysfunction, and oxidative stress play a crucial role in AF [15, 16]. As a sensitive indicator of the inflammation state in the body, hs-CRP levels are significantly increased in AF patients, suggesting that upregulated hs-CRP level is closely linked to AF pathogenesis [17]. Several previous studies have demonstrated the relatively high efficacy of statins in improving endothelial function and decreasing oxidative stress, while they also possess an anti-inflammatory and antithrombotic effect [18, 19]. More importantly, hs-CRP levels in AF patients treated with atorvastatin were lowered compared to the control untreated group, implying that atorvastatin suppressed inflammation by reducing the damage due to atrial electrical and structural remodeling, and prevented AF persistence, thereby reducing hs-CRP levels [20, 21]. Evidence, supporting the notion that atorvastatin therapy may impact hs-CRP levels in AF patients, is available [22, 23]; however, other studies contradict these findings [10, 21]. In order to address this issue, we used a meta-analysis approach and focused on the hs-CRP levels in AF patients before and after atorvastatin treatment.

## 2. Materials and Methods

### 2.1. Data Sources and Keywords

Bibliographic databases, (MEDLINE and EMBASE, Web of Science, Cochrane Library, PubMed, Google Scholar, China BioMedicine (CBM), and China National Knowledge Infrastructure (CNKI)), were exhaustively searched to identify published studies that assessed the change in hs-CRP levels in adult AF subjects who were administrated with atorvastatin. The search included studies available from the inception to June 2014. We used medical subject heading (MeSH) and keywords for atorvastatin and AF as follows: “atorvastatin” or “liptonorm” or “lipitor” and “Atrial Fibrillation” or “atrial fibrillations” or “fibrillation, atrial” or “familial atrial fibrillation” or “auricular fibrillation.” The search was limited to human studies and without restrictions to the language of the paper. In addition to the above electronic search, relevant articles were checked manually to identify additional potential papers.

### 2.2. Selection Criteria

This meta-analysis focused on observational studies where monitoring of hs-CRP was used to predict AF patients treated with atorvastatin. To be included in our meta-analysis, published studies fulfilled the following selection criteria: (1) patients with AF and the opportunistic screening for AF by pulse palpation, followed by recording of an electrocardiogram (ECG) to verify diagnosis [24]; (2) human-associated clinical trials focusing on AF and atorvastatin; (3) providing data on hs-CRP levels before and after atorvastatin medication; (4) providing information on the adjusted standard mean differences (SMDs) and 95% confidence intervals (CI) for hs-CRP level; (5) supplying the sample number; and (6) having sample sizes greater than 44. When the chosen studies included subjects that overlapped more than 50% in two or more studies, we only included the study whose sample population was the most comprehensive. Furthermore, only the most recent study of papers published by the same authors was included.

### 2.3. Data Extraction

In order to reduce the bias and enhance the confidence, two investigators separately extracted information from the selected studies based on the selection criteria and arrived at a consensus on all the items through discussion and reexamination. The following relevant data was extracted from the final selected studies for analysis: surname of first author, time of publication, source of publication, study type, study design, ethnicity and country of subjects, sample size, gender and age information, and detection method for hs-CRP levels in the human subjects. All authors approved the selected studies.

### 2.4. Quality Assessment

To determine whether the study in question was of high quality, two investigators independently scored the studies based on the Newcastle-Ottawa Scale (NOS) criteria [28]. The NOS criteria are as follows: (1) selection of the cohort: representativeness of the exposed cohort (NOS1), selection of the nonexposed cohort (NOS2), ascertainment of exposure (NOS3), and demonstration of the outcome of interest being not present at start of study (NOS4); (2) comparability of the cohort: whether the study was selected and analyzed according to the most important factor (NOS5) and whether the study controlled other confounding factors (NOS6); (3) assessment of outcome: follow-up long enough for outcomes to occur (NOS8) and adequacy of follow-up of cohort (NOS9). Discrepancies between the investigators on NOS scores were resolved by a third reviewer, through discussions with the two investigators. In addition, a validated tool for quality assessment of diagnostic accuracy, known as the Quality Assessment of Diagnostic Accuracy Studies (QUADAS), was applied to assess the methodological quality of the selected studies [29].

### 2.5. Statistical Analysis

Pooled odd ratios (ORs) with a 95% confidence interval (CI) were calculated, along with a* Z* test, to determine the effect size for each study. The ORs were calculated utilizing the STATA software, version 12.0 (Stata Corp., College Station, TX, USA) by two separate investigators. In order to supply quantitative data from all of the selected studies and to minimize the variance of the summary SMDs and 95% CI, we performed the current statistical analysis by utilizing a random-effects model (DerSimonian and Laird method) or a fixed-effects model (Mantel-Haenszel method). The random-effect model was applied when heterogeneity existed among the studies, while fixed-effects model was applied when there was no statistical heterogeneity. The heterogeneity across the enrolled studies was evaluated by Cochran's* Q*-statistic (*P* < 0.05 was regarded as statistically significant), and the degree of interstudy heterogeneity was measured by *I* ^2^ test (0%, no heterogeneity; 100%, maximal heterogeneity) [30, 31]. The metaregression and subgroup meta-analyses by country and detection method were performed to explore potential effect modification. Sensitivity analysis was performed by omitting single studies to test the reliability of the result. By visual inspection of the symmetry of Egger's funnel plot and assessment from the Egger's test, publication bias was further evaluated [32].

## 3. Results

### 3.1. Included Studies

**Figure 1:**
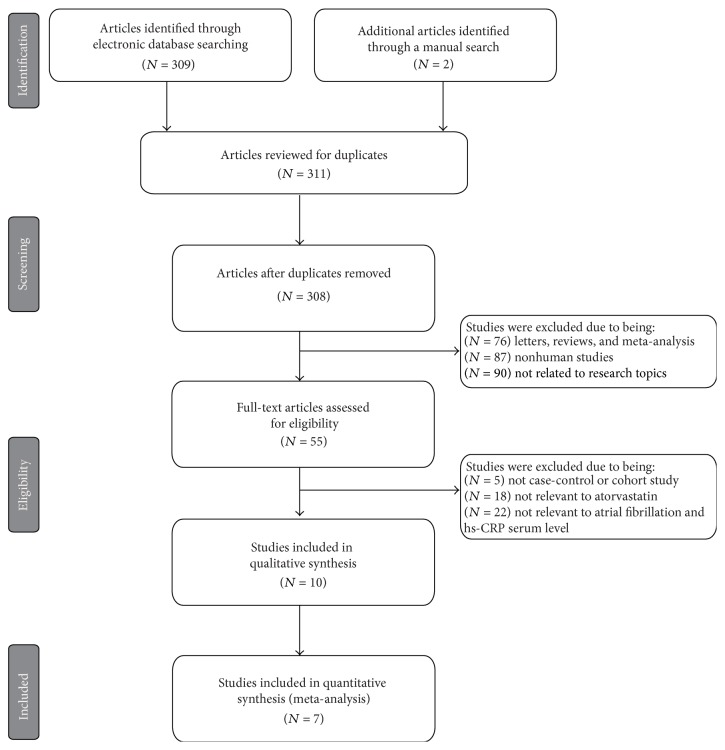
Flow chart of literature search and study selection. Seven clinical case-control studies were included in this meta-analysis.

**Figure 2:**
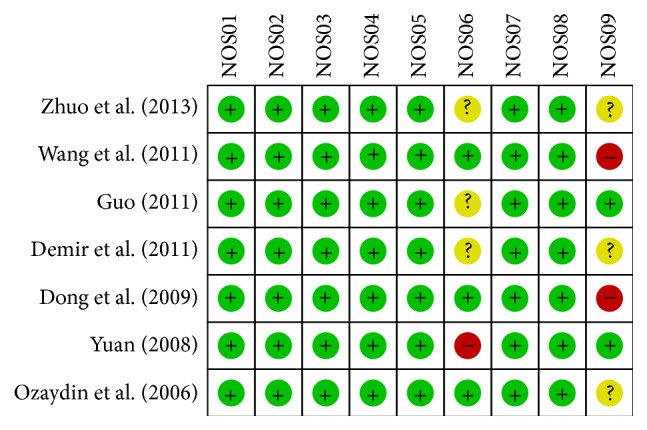
The methodological quality of included studies was evaluated by Newcastle-Ottawa Scale criteria.

Our present meta-analysis identified a total of 7 cohort studies, published between 2006 and 2013, that provided information on the changes in hs-CRP level in AF patients before and after administration of atorvastatin [10, 21–23, 25–27]. Demographic information, other characteristics, and methodological quality of the extracted studies included in our meta-analysis are presented in Table 1. All studies were performed in populations of Asian descent (*n* = 726) (369 patients were treated with atorvastatin and 357 patients with no statin treatment). The countries where the studies were performed were China (*n* = 5) and Turkey (*n* = 2). Gender data was also available from the majority of the included studies, with more males present in this meta-analysis than females; however, two studies [Wang (2011) and Guo (2011)] failed to report this information. The screening steps and study selection procedure are shown in Figure 1. Initially, a total of 311 papers were retrieved through the electronic database search and a manual search. Among those papers, three articles were duplicates and therefore excluded. Furthermore, letters, reviews, meta-analyses, nonhuman studies, and studies not related to the present research topics were excluded (*n* = 256). The remaining 55 studies were reviewed and an additional 45 studies were excluded since they were not case-control or cohort designed studies, not relevant to atorvastatin or not relevant to AF and hs-CRP levels. After the remaining 10 papers were fully reviewed, 7 papers were finally selected for our meta-analysis, and the other 3 articles were excluded for lack of data integrity. The quality scores of the studies were higher than 7 (high quality), as shown in Figure 2.

### 3.2. Change of hs-CRP Level in AF Patients

**Figure 3:**
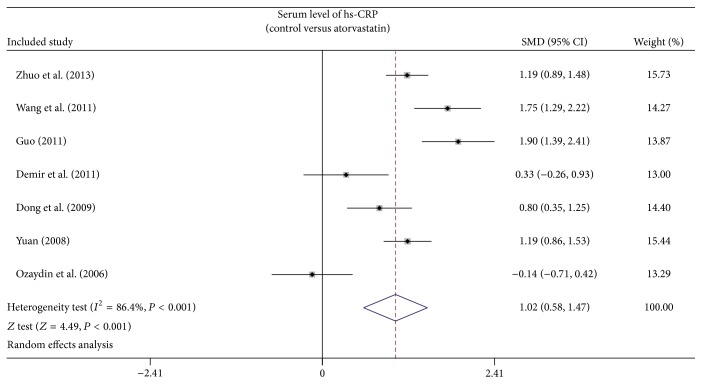
Forest plots for the change of hs-CRP level between AF patients and controls.

**Figure 4:**
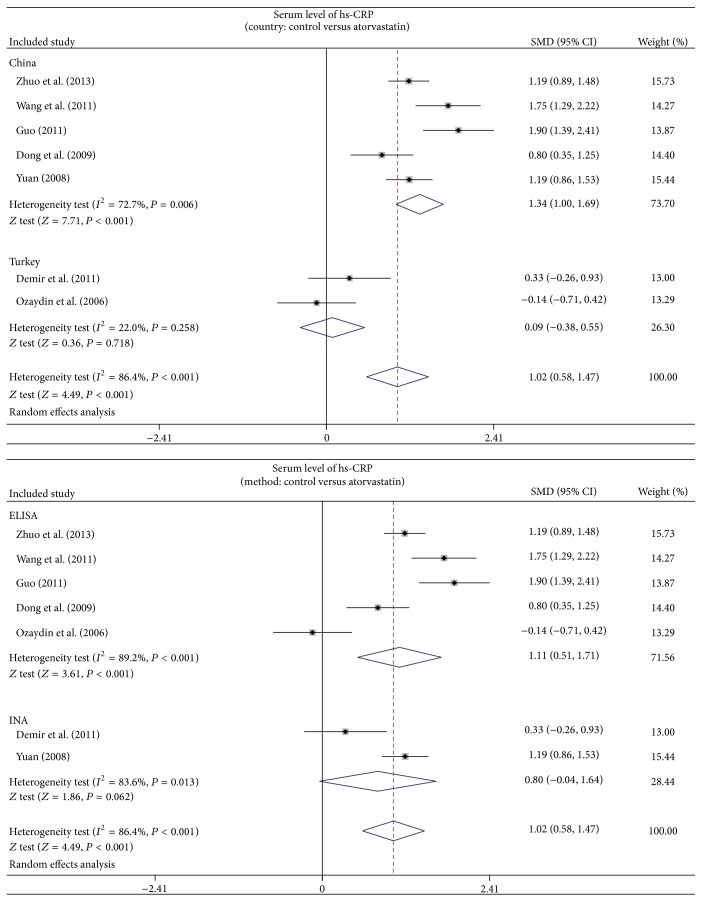
Subgroup analyses by country and method for the differences of hs-CRP level between AF patients and controls.

As shown in Figure 3, the results of our present meta-analysis revealed that after treatment with atorvastatin, hs-CRP level in AF patients decreased significantly (SMD = 1.02, 95% CI: 0.58–1.47, *P* < 0.001). Subgroup analysis based on country and detection methods showed that atorvastatin treatment reduced serum hs-CRP levels in AF patients in the Chinese populations (SMD = 1.34, 95% CI: 1.00–1.69, *P* < 0.001), and by using the ELISA detection method of hs-CRP level (SMD = 1.11, 95% CI: 0.51–1.71, *P* < 0.001), but not among Turkish populations (SMD = 0.09, 95% CI: −0.38–0.55, *P* = 0.718) and using the INA method for detection of serum hs-CRP (SMD = 0.80, 95% CI: −0.04–1.64, *P* = 0.062) (Figure 4).

### 3.3. Sensitivity Analysis and Publication Bias

**Figure 5:**
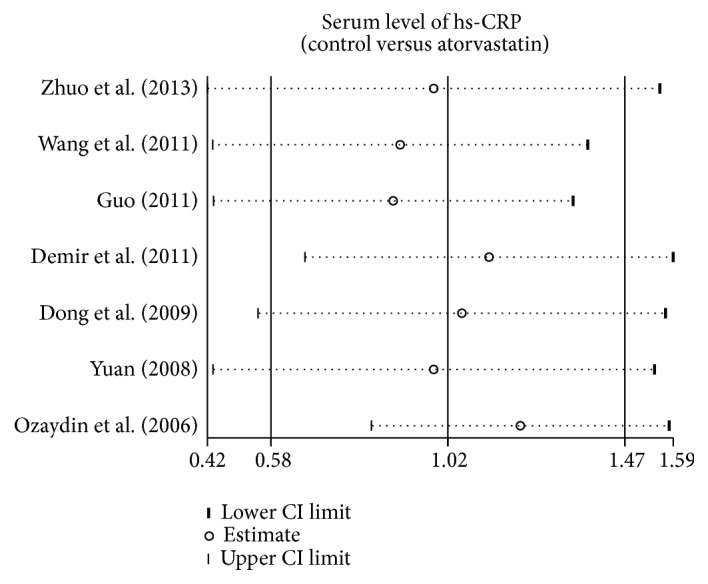
Sensitivity analysis of the summary odds ratio coefficients for the differences of hs-CRP level between AF patients and controls.

**Figure 6:**
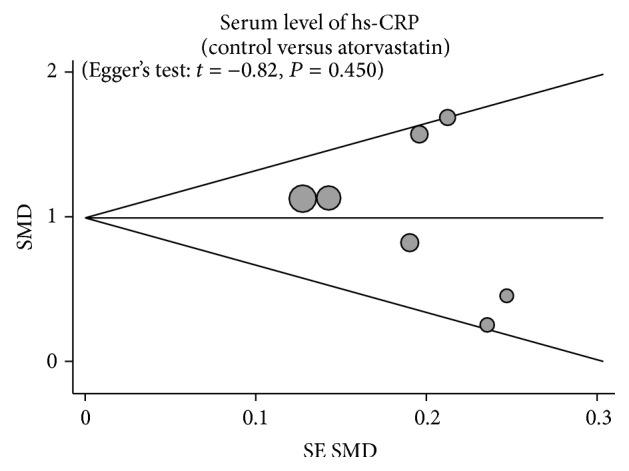
Funnel plot of publication biases for the differences of hs-CRP level between AF patients and controls.

Sensitivity analysis to evaluate the stability of the results was performed by removal of each study one by one. The corresponding pooled ORs in overall comparison and stratified analyses were not significantly altered, indicating a stable and credible outcome (Figure 5). As shown in Figure 6, no obvious visual asymmetry was observed from the graphical funnel plots and Egger's test, showing no publication bias (*t* = −0.82, *P* = 0.450).

## 4. Discussion

In order to investigate the relationship between atorvastatin treatment and the serum hs-CRP levels in AF patients, an extensive meta-analysis was undertaken, and our main result shows that a significant reduction in serum level of hs-CRP was achieved after atorvastatin treatment in AF patients. Statins inhibit HMG-CoA reductase and are used in dyslipidemia patients to reduce blood cholesterol levels and prevent cardiovascular diseases [33]. Statins influence inflammation and oxidative stress by regulating the expression of iNOS, TNF-*α*, and MMPs [7]. Furthermore, as an anti-inflammatory agent, statins improve endothelial cell function, inhibit vascular smooth muscle cell proliferation, stabilize atherosclerotic plaque, and reduce serum level of hs-CRP [34]. Atorvastatin, known widely as Lipitor, is effective as an antilipemic drug used to reduce lipoprotein-rich cholesterol, thus significantly lowering the risk of cardiovascular diseases [35]. Atorvastatin was also shown to influence the cellular activities of components of the PI3/AKT signaling molecules such as AKT, P2X7, pERK, RhoA, cyclin D1, and *β*-catenin to inhibit proliferation and induce apoptosis in pancreatic cancer cells [36]. Atorvastatin's cellular effect on proliferation and apoptosis was found to be beneficial in reducing the risk of development of advanced prostate cancer [37]. Additionally, atorvastatin suppressed the activation of ERK and AKT and disrupted Kras/PI3K and Kras/Raf complex formation in the NSCLC cells through its inhibitory action on HMG-CoA reductase [38]. It has been reported that atorvastatin treatment in AF patients, when accompanied by a reduction in serum level of hs-CRP, is associated with clinical improvement in AF patients [39]. After treatment with atorvastatin, AF patients recovered sinus rhythm and electrical cardioversion [7]. The lower CPR levels due to atorvastatin treatment suggest that atorvastatin helps in preventing remodeling of atrial electricity and structure and inhibits the inflammatory process to prevent the development of AF [21]. Atorvastatin could inhibit inflammation by lowering proinflammatory cytokine levels, such as that of IL-6 and hs-CRP, inhibiting the complementary system, and increasing the NO release from the endothelial cells [40]. As a result, atorvastatin is linked to reduction of endothelial cell interaction with neutrophils and other anti-inflammatory mechanisms, inhibition of degeneration and fibrosis of myocardial cells, and eventually limiting the occurrence of atrial structural remodeling in AF patients, suggesting an excellent therapeutic effect on AF through inhibiting inflammation [21]. Consistent with this, atorvastatin-treated animals also had lower hs-CRP levels and relatively shorter AF duration compared to the control group [20]. Based on our results, we propose that atorvastatin treatment decreased hs-CRP serum levels in AF patients due to the anti-inflammatory role of atorvastatin. In line with our conclusion, Höglund et al. also found a lower serum level of hs-CRP after atorvastatin treatment in the AF patient group compared to the placebo group over time [41].

To further analyze the influence of other related factors such as country and serum hs-CRP detection methods on the relationship between atorvastatin treatment and lower hs-CRP serum level, a subgroup analysis was performed. From the result of country-stratified analysis, we observed that the relationship was not affected in Chinese population but was not statistically significant in Turkish population. One possible explanation could be different life styles and genetic backgrounds of individuals from different countries. Based on the detection method for hs-CRP, the ELISA method did not affect the observed relationship between atorvastatin therapy and hs-CRP serum level. However, the INA method showed no statistically significant relationship and might be explained by technical differences between the different detection methods. In summary, our results of the effect of atorvastatin treatment in lowering hs-CRP serum levels in AF patients are in agreement with other studies, suggesting atorvastatin therapy is effective in AF patients.

There are several limitations in our study. First, we did not take into consideration the viral load or treatment efficacy of atorvastatin over time, and the long-term follow-up of patients was not completed. Second, our study was single-center, cross-sectional retrospective study, with relatively small number of articles and a smaller number of patients. This may have resulted in article and patient selection bias. Another restriction may be that we only evaluated the role of hs-CRP in AF patients treated with atorvastatin, while comparison with other anti-inflammatory indexes such as IL cytokines are lacking. Lastly, since only paroxysmal AF was analyzed, patients with persistent AF or postoperative AF may have responded differently to atorvastatin but were not investigated.

## 5. Conclusions

In conclusion, our meta-analysis investigated the hs-CRP levels in AF patients treated with atorvastatin. We observed that hs-CRP levels decreased significantly in AF patients who used atorvastatin during the study period. Nevertheless, this study did not follow up patients over time, so the predictive values of hs-CRP levels may be limited. Therefore, further investigation is necessary.

## Figures and Tables

**Table 1:** Main characteristics and methodological quality of eligible studies.

First author	Year	Country	Ethnicity	Total	Sample size		Gender (M/F)		Age (days)		Disease	Method	NOS
					Atorvastatin	Control	Atorvastatin	Control	Atorvastatin	Control			
Zhuo [22]	2013	China	Asians	206	104	102	61/43	55/47	64.0 ± 10.0	61.0 ± 13.0	AF	ELISA	8
Wang [25]	2011	China	Asians	98	50	48	—	—	55.6 ± 10.7	55.6 ± 10.7	PAF	ELISA	6
Guo [23]	2011	China	Asians	87	45	42	—	—	60.2 ± 8.3	60.2 ± 8.3	PAF	ELISA	6
Demir [21]	2011	Turkey	Asians	44	22	22	10/12	13/9	62.0 ± 9.0	60.0 ± 10.0	PAF	INA	7
Dong [26]	2009	China	Asians	81	41	40	25/16	26/14	56.0 ± 15.2	56.0 ± 15.2	AF	ELISA	7
Yuan [27]	2008	China	Asians	162	83	79	50/33	51/28	58.0 ± 11.2	58.0 ± 11.2	AF	INA	8
Ozaydin [10]	2006	Turkey	Asians	48	24	24	17/7	12/12	61.0 ± 13.0	64.0 ± 9.0	AF	ELISA	7

M: male; F: female; NOS: Newcastle-Ottawa Scale; PAF: paroxysmal atrial fibrillation; AF: atrial fibrillation; ELISA: enzyme linked immunosorbent assay; INA: immunoturbidimetry.
